# Corrigendum to “Challenges in Interpretation of Thyroid Function Tests in Pregnant Women with Autoimmune Thyroid Disease”

**DOI:** 10.1155/2017/4324130

**Published:** 2017-10-25

**Authors:** Ulla Feldt-Rasmussen, Sofie Bliddal, Åse Krogh Rasmussen, Malene Boas, Linda Hilsted, Katharina Main

**Affiliations:** ^1^Department of Medical Endocrinology, Rigshospitalet, Blegdamsvej 9, 2100 Copenhagen, Denmark; ^2^Department of Growth and Reproduction, Rigshospitalet, Blegdamsvej 9, 2100 Copenhagen, Denmark; ^3^Department of Clinical Biochemistry, Rigshospitalet, Blegdamsvej 9, 2100 Copenhagen, Denmark

In the article titled “Challenges in Interpretation of Thyroid Function Tests in Pregnant Women with Autoimmune Thyroid Disease” [1], the name of the second author was given incorrectly as Anne-Sofie Bliddal Mortensen. The author's name should have been written as Sofie Bliddal. The revised authors' list is shown above.
